# Molecular dynamics simulation on surface modification of quantum scaled CuO nano-clusters to support their experimental studies

**DOI:** 10.1038/s41598-022-16751-w

**Published:** 2022-10-05

**Authors:** Adil Loya, Jacqueline L. Stair, Farid Uddin, Guogang Ren

**Affiliations:** 1 Department of Mechanical Engineering, National University of Sciences and Technology, H-12, Islamabad, Pakistan; 2 School of Life and Medical Sciences, University of Hertfordshire, Hatfield, AL10 9AB UK; 3 College Lane, School of Engineering and Technology, University of Hertfordshire, Hatfield, AL10 9AB UK

**Keywords:** Engineering, Fluids

## Abstract

Interest in nanoparticle modification using functional chemicals has increased rapidly, as it allows more freedom of physiochemical tuning of the nanoparticle’s surface into biomedically oriented and designated properties. However, the observation and detection of the thin molecular layers on the nanoparticle surface are very challenging under current analytical facilities. The focus of this research is to demonstrate fundamental interactions between the surface treated nanoparticles and their host liquid media using lab-based experimentation and simulation. In this research, investigation has been carried out on analyzing the surface compatibility and the diffusivity of modified CuO nanoparticles (CuONPs) with short-chain carboxylate-terminated molecules in biofluids. Moreover, during the current Covid-19 pandemic, the Cu/CuONPs have proved effective in killing SARS-CoV1/2 and other airborne viruses. This research was conducted at the molecular level with joint consideration of experimental and simulation studies for characterization of variables. Experimental tests conducted using Fourier Transform Infrared (FTIR) spectroscopy demonstrated several spectral ranges of interest, specifically, detection of three major carboxylate attachments (i.e., 1667–1609 cm^−1^, 1668–1557 cm^−1^, etc.) were found. From simulation, similar attachment styles were observed by the LAMMPS simulation package that mimicked similar agglomerations with a predicted diffusion coefficient as recorded to be 2.28E−9 m^2^/s. Viscosities of modified nanofluids were also compared with unmodified nanofluids for defining aggregation kinetics.

## Introduction

Metal oxide nanoparticles (MONPs) such as Copper oxide (CuONPs) are increasingly used in applications such as fillers, inks, opacifiers, catalysts, semiconductors, cosmetics, and microelectronics^1–3^. CuONPs have  wide potential for industrial applications such as gas sensors, catalytic processes, and solar energy photocatalytical as well as antiviral and antibacterial applications in healthcare settings^3–5^.

One of the CuONPs challenge in biological applications is their tendency of agglomeration and dispersion instability in the liquid suspension^6^ using various dispersing devices^7^. Studies show that the CuONPs in a fully dispersed state can be used in a number of applications where the thermal conductivity, overall tribological performance^8^, electrochemical activities (i.e., in the hydrogen fuel cells)^9^ were all improved. Other significant improvements include enhanced property of magnetic disks (CuO nanowire had low ferromagnetism behavior)^10^, inkjet printers, and smoothed electrospinning processes for fiber production^11^.

Short-chain carboxylate attachments and amine group attachments are frequently being used for the modification of surfaces for better and stable dispersions. For example, some studies show that the amine group surface modification of CuONPs can be beneficial for biological systems by improving their optical properties, such as fluorescence and fluorescence decay^12^. Meanwhile, some long-chain carboxylate group was also used for surface modifications for supporting the dispersion of nanoparticles in polarized solvents such as water^13^. Furthermore, Soleimani and Taheri investigated lipophilic properties of CuONPs in various polar solvents and concluded that the highest dispersion is shown in CCl_4_^14^.

BA as a multifunctional small molecule, which has shown promise in the surface treatment of nanoparticles including CuONPs because of its wide range utilization for biomedical and healthcare setting applications. In this work, we focus on the modifications of the CuONPs using BA, which was then modelled and simulated by LAMMPS and validated using experimental results. Previously, a study also showed that butyrate acids are potent surface treatment agents for the anti-inflammatory therapy of ulcerative colitis when used with solid lipid nanoparticles^15^.

It has also been found that butyrate or short-chain fatty acids help as a source of energy for the colon mucosa by stimulating fluid and electrolyte absorption, thereby preventing colon inflammation, and regulating the colon’s defence barriers^16^. Butyrate groups can also act as a chemo-preventive agent^17^ in an in-vitro exposure of tumour cells, which showed induced apoptosis and inhibition of proliferation. Therefore, due to these factors, BA was chosen as a modifying agent for the nanoparticles serving as the carrier particles for drug-delivery^18^. Nevertheless, CuO already acted as an antibacterial and antiviral agent, and the additional surface treatment using BA added a further enhancement of inhibiting both tumor cells and bacteria due to the presence of butyrate^18^.

This mechanism can be applied to other MONPs for application in the biomedical field^19^. For instance, if super magnetic iron oxide nanoparticles were functionalized using carboxylate group, they will highly dislocate the biofilms and inhibited the growth of *S. aureus* compared to untreated biofilms (by over 35% after 24 h). Thereby, functionalizing of the MONPs can be extremely optimistic for enhancing biodegradability and antibacterial activity by carboxylate modification. Other research showed Gallium oxide nanoparticles were modified by using carboxylate groups (i.e., COOH) and amine groups (i.e., NH) to inhibit the bacteria^20^. Therefore, it can be concluded that the studies of the carboxylate functional group on nanoparticle surface can facilitate a high rate of inhibition.

Moreover, the butyrate group shows inhibitive activity towards tumor cell growth, i.e., it acts as histone deacetylase inhibitor and provides anti-neoplastic properties^21^. In addition to this Tomy J. Gutierrez and Vera A. Alvarez used butyrate as a functional group with thermally stable ferric nanoparticles for paclitaxel carrier applications^22^. The current utilization of butyric chains with TiO_2_ nanoparticles and some noble metal ions under UV irradiation has shown promising byproducts such as H_2_, propane and propene generation as investigated by Scandura et al.^23^. Xue et al. investigated the usage of butyric chain ligands with Pd nanoparticles, to modulate the catalytic reaction process and optimize the stability of reaction^24^.

In this study, CuONPs have been modified with BA as a model system and molecular dynamics simulation has also been performed for analyzing diffusivity, level of agglomeration, aggregation dependent on viscosity and interaction kinetics. By doing so, experiments were validated by the simulation visualizations. Currently, this work is used to estimate the bonding distance and properties with BA functional groups.

Today, in the current Covid-19 pandemic, the Cu/CuONPs have proved effective in killing SARS-CoV1/2 and other airborne viruses, as investigated by Ren and Lin in their latest research outcomes conducted in 2021^25^. Therefore, by conducting simulations on CuO dispersion in aqueous medium with a surfactant (that holds properties of inhibition) can help in understanding the inhibiting science of these modified particles and how it degrades the production of viruses. The virus disinfectant properties of CuONPs by formation of Reactive Oxygen Species (ROS) defines why CuO is a best candidate for antiviral applications. CuONPs impregnated masks have been proven to be protective against anti-influenza virus (i.e., H1N1 and H9N2)^26^.

**Figure 1:**
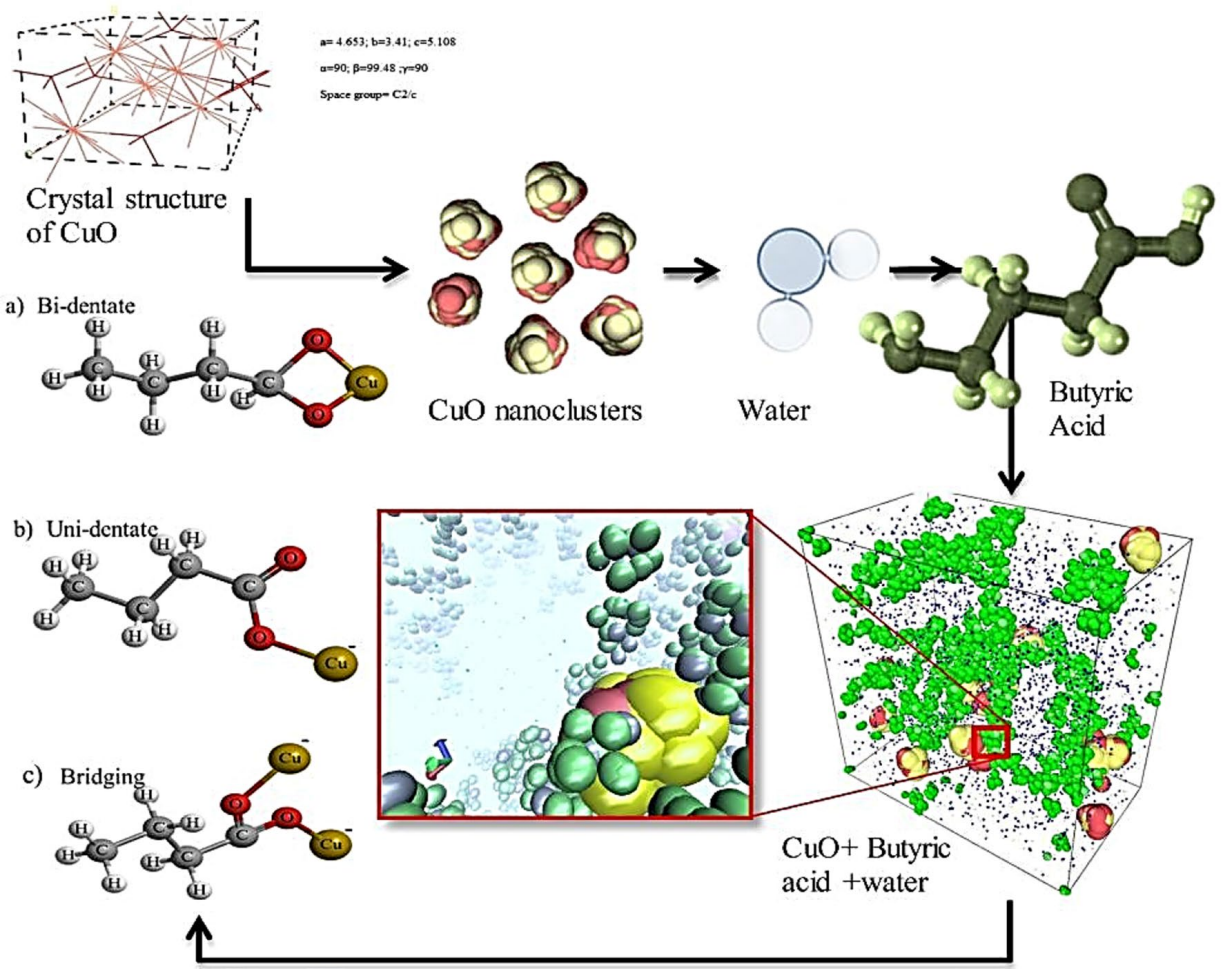
Schematic of molecular dynamics procedure for modification of CuO nanoparticle with BA.

Therefore, this simulation can act as an introductive point for enabling other researchers simulating viruses with such systems to identify the efficacy of functional nanofluid over different evolving viruses. In addition to this, the work holds novel standing as it is hard to find molecular dynamics simulation work on CuONPs modified with BA or short-chain carboxylate groups. A novel approach for simulating metaloxide nanofluids with surfactant has been demonstrated. Aggregation dynamics are discussed on the basis of molecular dynamic simulations for defining the mobility of the surface modified particles in aqueous suspension. Nevertheless, the applications of modifying the nanoparticles with short chain fatty acids are many as already stated in the literature. BA modification helps in finding novel methods to entangle the nano-agglomeration mechanism and to understand the further formation of several carboxyl groups at sub-nanoscale range. In this study, radial distribution functions will be used for predicting several carboxyl groups using molecular dynamics, which will then be compared with FTIR results attained from lab-based experimentation. Moreover, the viscosities have been analyzed using experiments and simulations to know behavior of fluid interaction with nanoparticles. Figure 1, represents the procedure followed for performing molecular dynamics simulation for surface modification of CuONPs using BA.

### Materials and methodology

**Figure 2:**
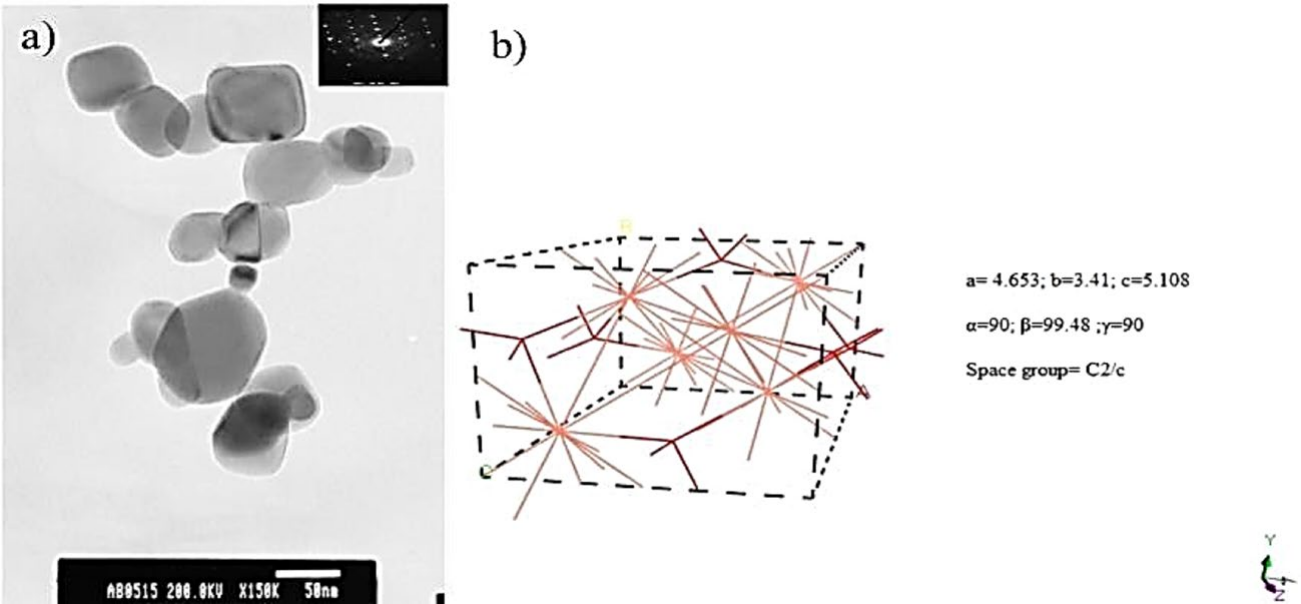
CuO nanoparticle image (**a**) observed by TEM. The average size of the nanoparticles is approximately 50 nm (Xu et al.^27^). The CuO crystal structure is shown in the upper-right corner, (**b**) CuO++ structure and its structural properties produced by Material Studio.

The CuONPs were obtained from QinetiQ materials with a density of 6.30–6.49 g/cm^3^^27^ and particle size from 10 to 70 nm as shown in Fig. 2a, the CuONPs was observed and analyzed using standard Transmission Electron Microscope (TEM).

CuONPs were used for surface modification. Equivalently, equal mole ratio with 1.5 g of BA was used as a surface treatment agent, which was obtained from Sigma Aldrich. 1 M solution of NaOH purchased from Sigma Aldrich was used for experimentation. For sonication the ultrasonic probe from Heilscher SP200 was used for dispersing nanoparticles into fluids before refluxing the mixture. The nanoparticles with surface treatment were also analyzed by Nicolet Thermoscientific Fourier Transform Infra-Red (FTIR) 8700 spectrometer. Furthermore, the crystal structure of CuONPs was built on Material Studio as shown in Fig. 2b. Some properties of the nanofluid are given in the nanofluid properties Table 1. Experimental viscosities were analyzed by TA 500x rheometer by TA instruments.

**Table 1:** Nanofluid and surfactant properties.

**Properties**		
Molar ratio	1:1000	
CuO Nps	0.2 g	Density = 6300 kg/m^3^ from QinetiQ
Butyric acid	1.5 g	Density = 946 kg/m^3^ from Sigma Aldrich
Dispersion temperature	90 °C	Refluxed
Reaction time set for first experiment	2 h	pH 7
Reaction time set for second experiment	24 h	pH 7
Reaction time set for third experiment	2 h	pH 5.5

### Surface modification of CuO nanoparticles by using butyric acid

The surface of CuONPs was modified using BA. Three different modifications were carried out: (1) At pH 7, the CuO-NPs were modified using BA, reaction lasted for 2 hours, (2) at pH 7, allowed the reaction to last for 24 hours, and (3) at pH 5.5, carried out the reaction for 2 hours.

#### Modification 1

The nanoparticles were initially dispersed in the BA at pH7, the suspension was vigorously stirred, then was sonicated for 10 min, and finally the solution was refluxed for 2 h. Specifically, 1.5 g BA was neutralized to pH7 using 1 M NaOH. Then, 0.2 g of CuO NPs were added to the mixture and sonicated for 10 min with vigorous stirring to form a dispersion. The dispersion was then heated and refluxed at 90 °C and stirred for 2 hours. The molar equivalence for the above mixture was 1:1000.

The suspension mixture was cooled and centrifuged at 8000 RPM for 20 minutes. Addition of Ethanol (approx. 10–20% v/v of reaction mixture) was added for settling the derivatized particles, then the supernatant was discarded. The obtained solid was re-dispersed into 10–20% ethanol/water solution through sonication, and then centrifuged again as described above. This process of Water/EtOH washing was repeated three times followed by an additional three washes by using a 100% EtOH. Finally, the solid was dried.

#### Modification 2 and 3

The same procedure was used for the second modification, however, with a refluxing condition of 24 hr was set.

For the preparation of modification 3, the same procedure was used, and the pH was reduced to 5.5 using NaOH (1 M).

### FTIR characterization of surface modified CuO nanoparticles

Chemically, the surface modified CuONPs were analyzed using Fourier Transform Infra-Red (FTIR) spectroscopy, specifically a Nicolet Thermoscientific FTIR 8700. The results were obtained using transmission mode under lab air environment. A control or reference spectrum of CuONPs was also collected at the same time. FTIR spectra within the range of 7800–350 cm^−1^ were compared between the BA chemically modified CuONPs and the pure CuONPs.

### Simulation methodology

**Figure 3:**
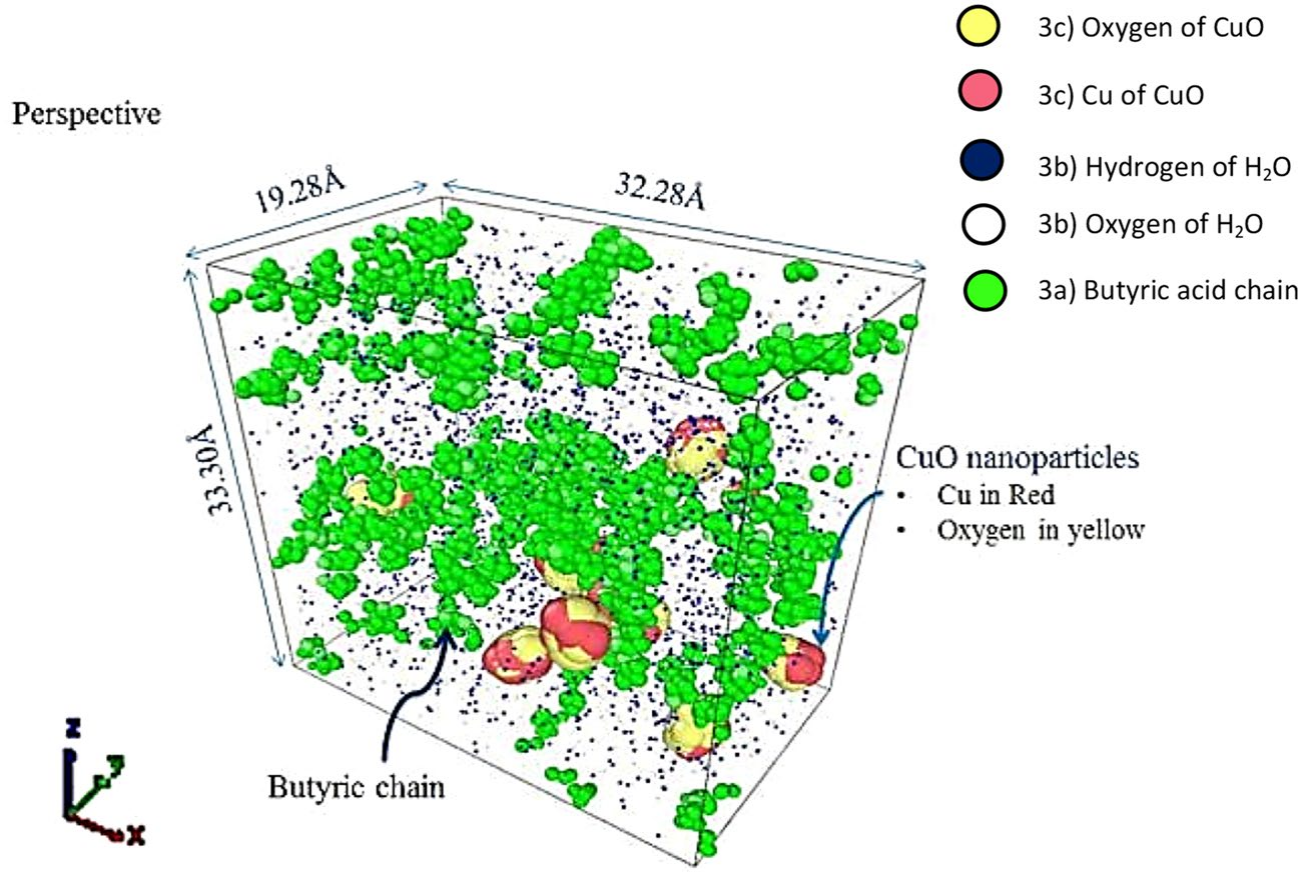
Simulated visualization of the nanoparticles with functional groups in a water system; (**a**) Green indicates the alkyl chain; (**b**) dark navy blue colour represents hydrogen and white represents oxygen; (**c**) Yellow–Red particles bonded together are CuO nanoclusters.

The simulation was performed using LAMMPS molecular dynamics software (LAMMPS MDS) with a courtesy of Plimpton^28^. The simulation cell dimension was of 32.28 Å × 19.28 Å × 33.3 Å in geometry as shown in Fig. 3. The simulation was performed using COMPASS force field^29–31^, to attach molecules and the simulation was carried out for approximately 16 picoseconds. For applying charges on different atoms, the COMPASS forcefield was used, before the simulation was performed using LAMMPS MDS. Charges set on atoms are shown in Table 2. The input atomic file is designed with the charges that were utilized during simulation. The charges incorporated using a Compass forcefield on different atoms are given in Fig. 3.

**Table 2:** Charges set on atoms.

Atom type	Charges	Atom type	Charges
C1	− 0.106	*H7*	0.516
C2	0.108	*H8*	0.159
C3	− 0.105	*H9*	0.569
C4	− 0.265	*HH1*	0.41
Cu	0.8	*HH2*	0.41
H10	0.106	*O*	− 0.8
H11	0.106	*O1*	− 0.82
H12	0.053	*O5*	− 0.73
H13	0.159	*O6*	− 0.98
H14	0.41	*OH*	− 1.23

**Figure 4:**
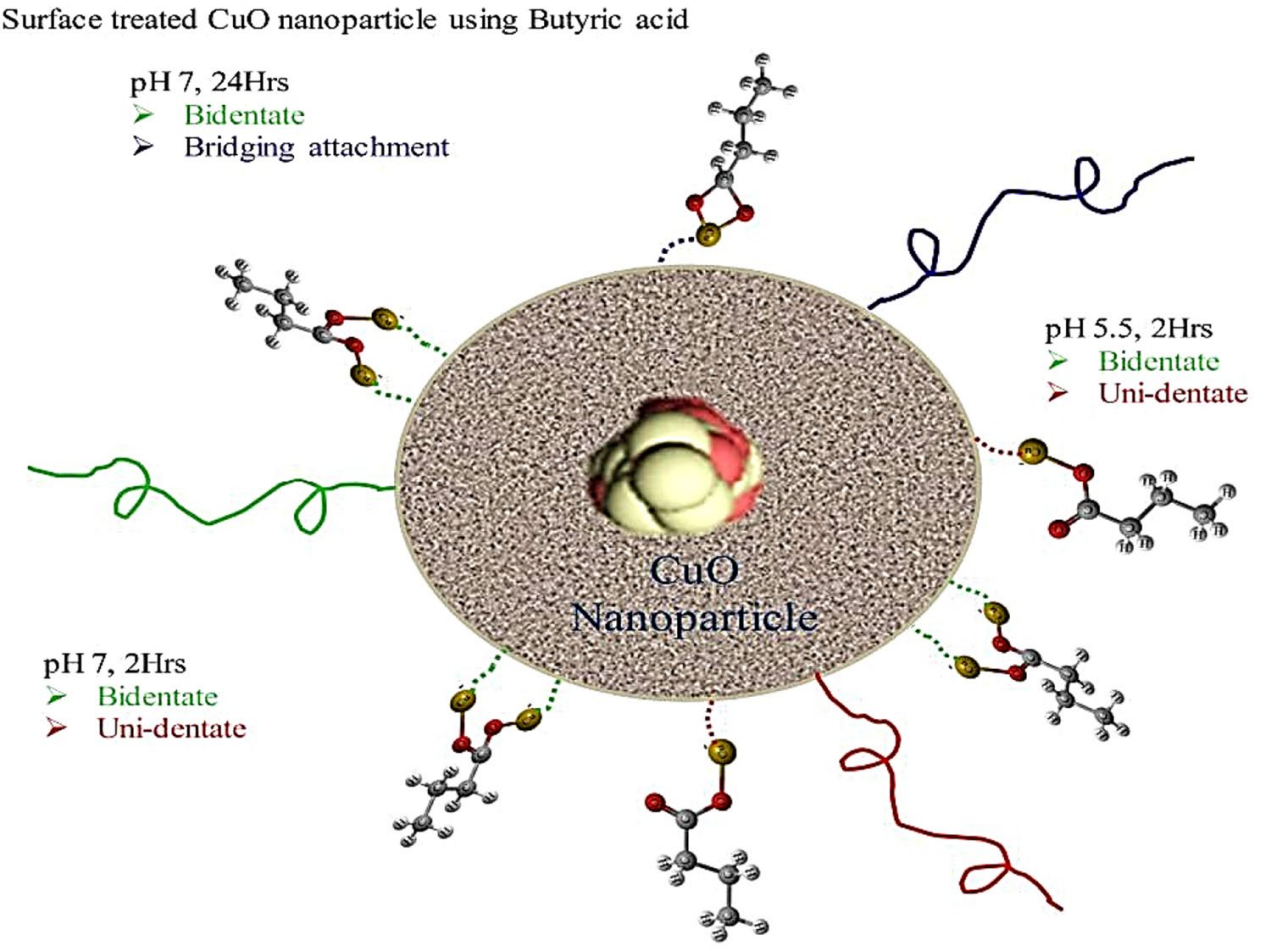
Schematic representation of the surface functionalization of CuO nanoparticles using butyric acid; this image shows various attachment styles found by changing reaction conditions.

Then dynamics simulations were performed using Discrete Particle Dynamics (DPD) and Smoothed Particle Hydrodynamics (SPH) potentials^32^. The DPD incorporates the Brownian dynamics and forces that govern random motion in the system, whereas the SPH governs the hydrodynamic effect of the fluid. The RDF results have been used in predicting the surface functionalization of carboxylate moieties on the surface of CuO nanoclusters. The whole setup is illustrated in a schematic representation as shown in Fig. 4, indicating how the functionalization was carried out and what was obtained after functionalization. Concentration of nanoparticles was about 1.9 volume% with 463 tip3p water molecules and the volume % used in experiments was about 1.5–2%. Simulation was setup with periodic boundary conditions. Simulations were performed using the NVT ensemble, which ensures that the system volume is consistent during the performance of the simulation. Velocity dist Gaussian technique was used to perform kinetics within the system. The randomness of the velocity factor was controlled by the fluctuation of temperature. Within the simulation system (box), a size cutoff was set to about 2.0 Angstrom. This simulation box was initially equilibrated for more than 50,000 steps followed by three different temperature simulations i.e., from 303 to 323 K with an increment of 10 K. Interactions between the molecules were calculated using radial distribution function using Eq. (1), where r is the radius between the atoms; ρ is the number density of the system, dr is the distance from next atom and 4πr^2^dr defines the volume of spherical shell.

1 $$g\left( r \right) = \frac{dn\left( r \right)}{{4\pi r^{2} dr\;\rho }}.$$

## Results and discussion

**Figure 5:**
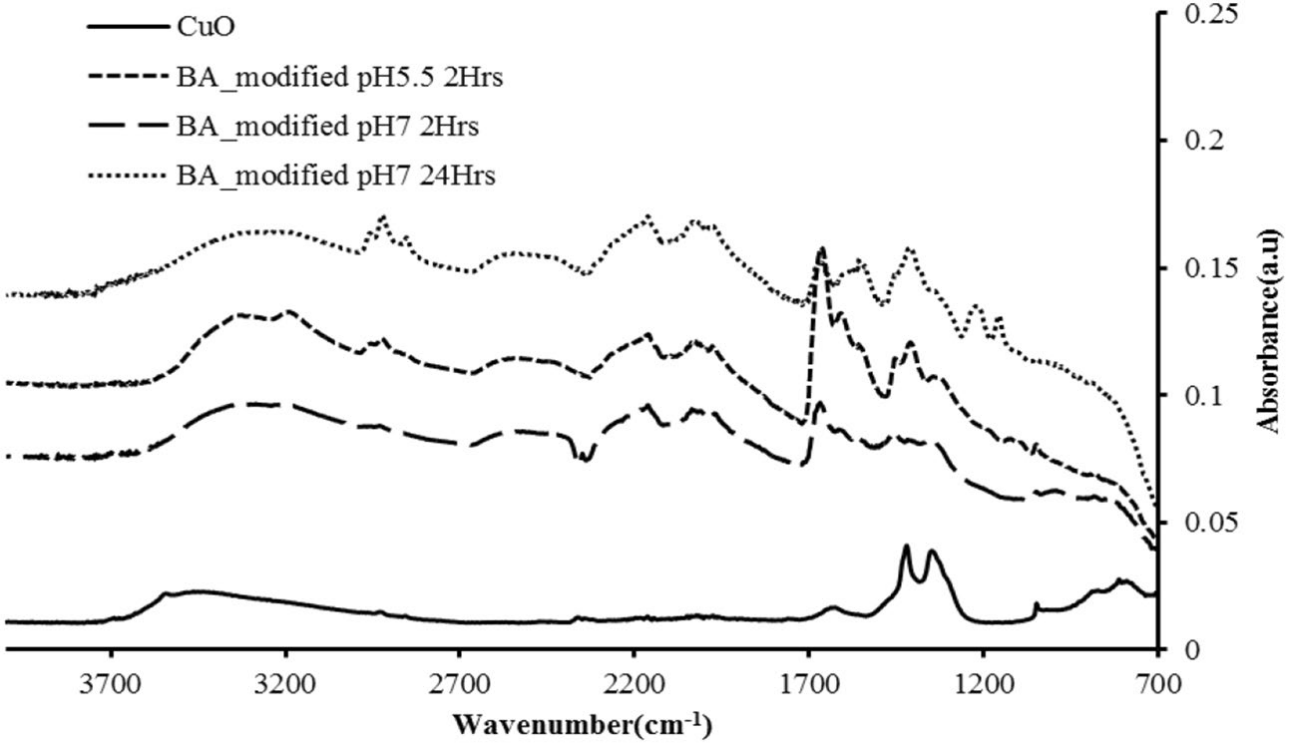
Modified CuO nanoparticles by using butyric acid as the reagent; (**a**) CuO nanoparticle original particles; (**b**) CuO nanoparticle modified using BA (Reaction time 2 h and pH 5.5); (**c**) CuO nanoparticle modified using BA (Reaction time 2 h and pH 7); (**d**) CuO nanoparticle modified using BA (Reaction time 24 h and pH 7).

The surface modification resulted in carboxylate attachment, in the form of either unidentate or bidentate, to the surface of CuONPs as shown in Fig. 5. The FTIR spectra of the modified nanoparticles indicates three types of targeted samples. This phenomenon and related structures, is demonstrated in Fig. 4, simply for visualization of a single CuONP surrounded by BA chains in which the polarized parts are connected to the CuONPs. Moreover, the unidentate and bidentate carboxylate attachment on CuONPs is dependent/exchangeable accordingly based on the suspension pH value as well as the time of reaction, which was either for 2 or 24 hr, respectively. Furthermore, different peaks along with the wavelength produced uni-dentate, bi-dentate or bridging attachment as shown in Table 3.

**Table 3:** Surface modification of CuONPs using BA, using different conditions, and the FTIR peak differences.

Attachment style	Surface treatment condition and reaction time		Peak difference
Bidentate	(a)	pH 5.5, 2 h	(a) 1667–1609 = 58 cm^-1^
Attachment	(b)	pH 7, 2 h	(b) 1456–1352 = 104 cm^-1^
	(c)	pH 7, 24 h	(c) 1668–1555 ~ 110 cm^-1^
Uni-dentate	(a)	pH 5.5 time = 2 h	(a) 1609–1410 ~ 200 cm^-1^
Attachment	(b)	pH 7 time = 2 h	(b) 1609–1456 = 153 cm^-1^
Bridging attachment	(c)	pH 7 time = 24 h	(c) 1555–1410 = 145 cm^-1^

First column listed the predicated attachment chemical structures under different conditions and 3 types of peaks difference achieved providing details for the BA attachment structures.

The surface modified CuONPs showed carboxylate attachments covering a large wavenumber range, i.e., approx. 1300–1750 cm^−1^. This range is related to the type of carboxylate group attachment that is also reported by Jian et al.^33^.

### FTIR analysis

**Figure 6:**
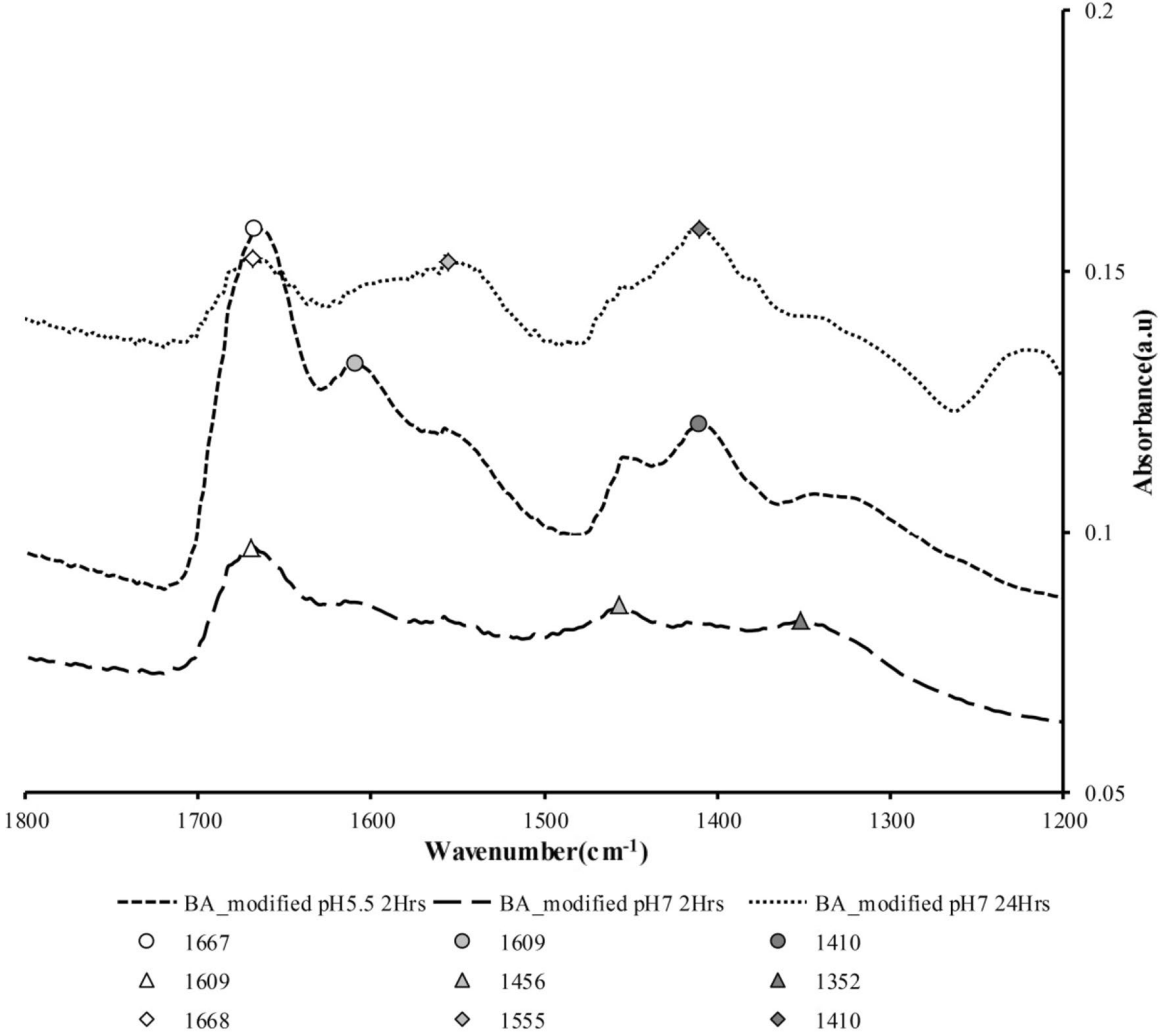
Modified CuONPs by using butyric acid as the reagent (**a**) open circle BA_modified at pH 5.5 for 2 hours, (**b**) open triangle BA_modified at pH 7 for 2 hours and (**c**) open diamond BA_modified at pH7 for 24 hours.

The FTIR spectrum of experiment 1 shown in Fig. 6 demonstrates the attachment of BA functional group to CuONPs at pH5.5 with 2 hours of reaction time. The possible attachments of uni-dentate or bi-dentate were reflected by their peak difference. If the difference is less than 110 cm^−1^, it is likely a bi-dentate attachment and if it is more than 200 cm^−1^ it becomes a uni-dentate attachment, while if the range is between 140 and 200 cm^−1^, it is likey a bridging ligand^33–35^. Table 3 listed the different peaks ranging from 1667 to 1609 cm^−1^ (~ 58 cm^−1^) i.e., bi-dentate attachment to the CuO nanoparticles. But at the same time, it also shows the uni-dentate attachment in which the two peaks give a difference of approximately 200 cm^−1^ between 1609 and 1410 cm^−1^.

The FTIR spectrum from experiment 2 shown in Fig. 6 represents the difference of 104 cm^−1^ for the peaks from 1456 to 1352 cm^−1^, which represents bi-dentate attachment to the CuONPs. However, at the same time it also shows the uni-dentate attachment, since when the difference of two peaks, i.e., from 1669 to 1456 cm^−1^ is taken, it gives approximate difference of 213 cm^−1^. It shows uni-dentate attachment to the CuONPs, so the predicted structures for both the attachments are shown in Fig. 6.

**Figure 7:**
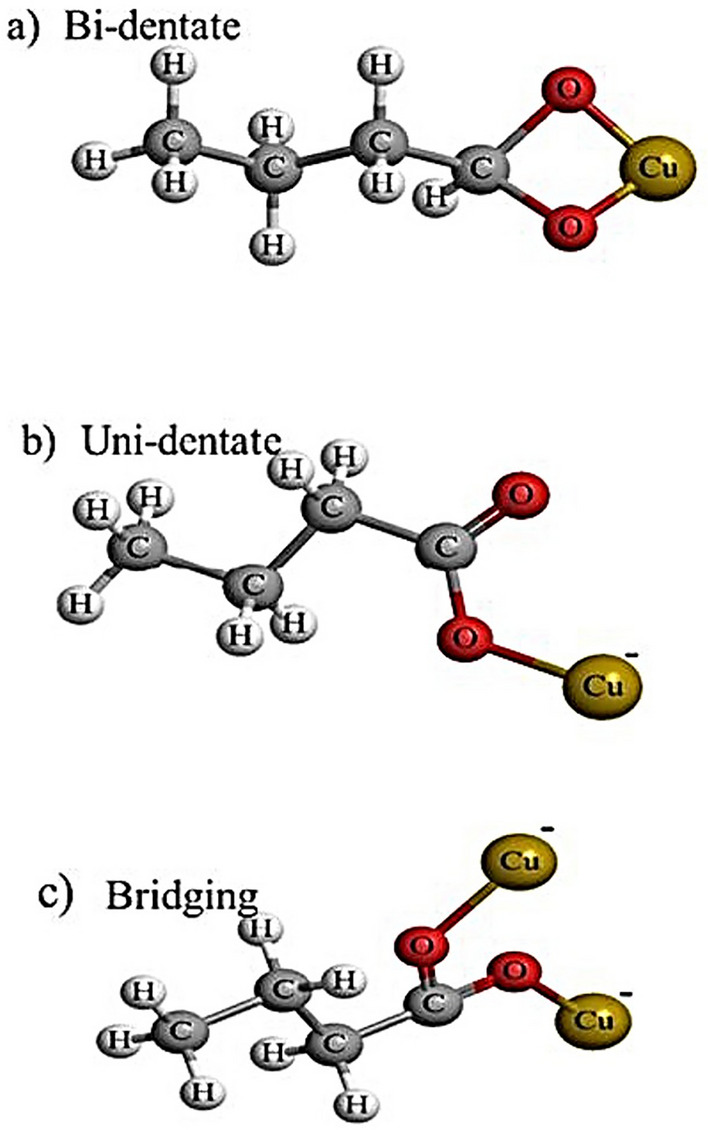
Different ligand attachment style on the surface CuO nanoparticles.

The FTIR spectrum from experiment 3 shown in Fig. 6 represents the difference of 145 cm^−1^ for peaks from 1555 to 1410 cm^−1^, which represents bridging attachment to the CuONPs. However, at the same time it also shows the bi-dentate attachment as when the difference of two peaks, i.e., from 1668 to 1557 cm^−1^ is taken, it gives approximate difference of 110 cm^−1^, so it shows uni-dentate attachment to the CuONPs; hence the predicted structures for both the attachments are shown in Fig. 7 respectively.

### Simulation on surface modifications

Simulation results were obtained from LAMMPS and visualized using Ovito. The suspension aggregation was observed while the functional group of BA was being attached to the surface of nanoparticles. In contrast to experimental modification, similar aggregations were recorded on the simulation criteria. These characterized results defined the agglomeration mechanism of the CuONP in water due to the effective attachment chemistry of the carboxylic group. However, on the simulation platform, different attachment styles were observed during various time scales of simulation phase.

**Figure 8:**
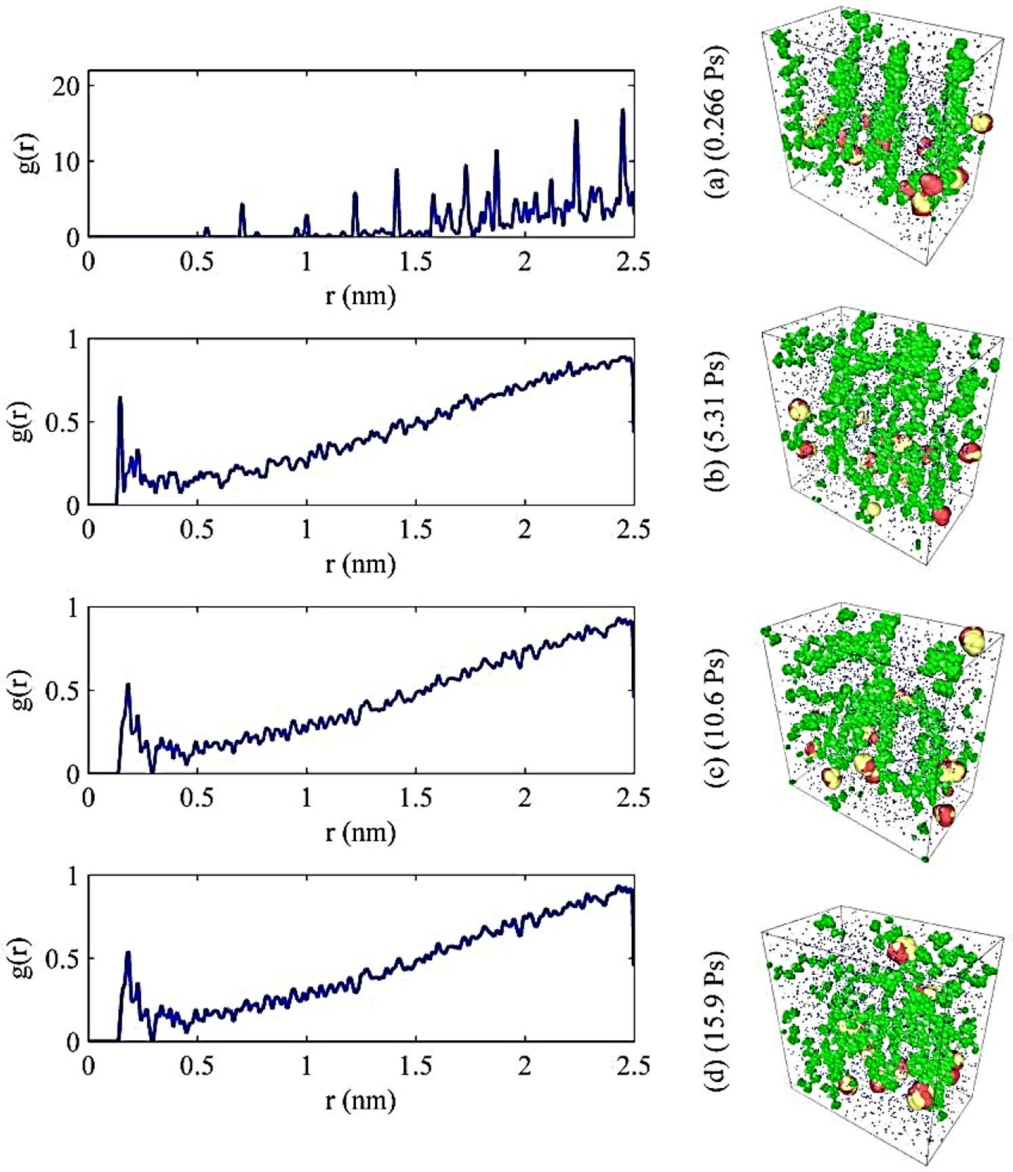
Radial distribution functions of sub-nanometer CuO with BA in water system.

The first characterization was the visual interpretation of the data based on the graphical images visualized by the simulation output. The graphical images were explained by the radial distribution function (RDF). This function elaborates on the particle–particle interactions and the agglomeration taking place between the particles. This RDF can also be backed using the diffusion coefficient. The RDF results shown in Fig. 8 show the agglomeration effect and on the right-hand side is the image representing the agglomerated effect of nanoparticles due to the surface modification of CuO nanoclusters using BA. From Fig. 8, it can be predicted that the attachment of the CuO and butyric chains causes high agglomeration since the spacing between the molecules is less i.e., its strongly peaking at 0.1–0.15 nm from Fig. 8b–d. Further, at 2 nm a small but stretched peak can be seen that could be due to the harmonic movement of some distant molecules in the system. In Fig. 8a, the nanoclusters are not agglomerated in contrast to Fig. 8b–d, since Fig. 8a shows the initial stages of simulation; i.e., the system has just started to equilibrate, and it has not reached the desired equilibration time.

**Figure 9:**
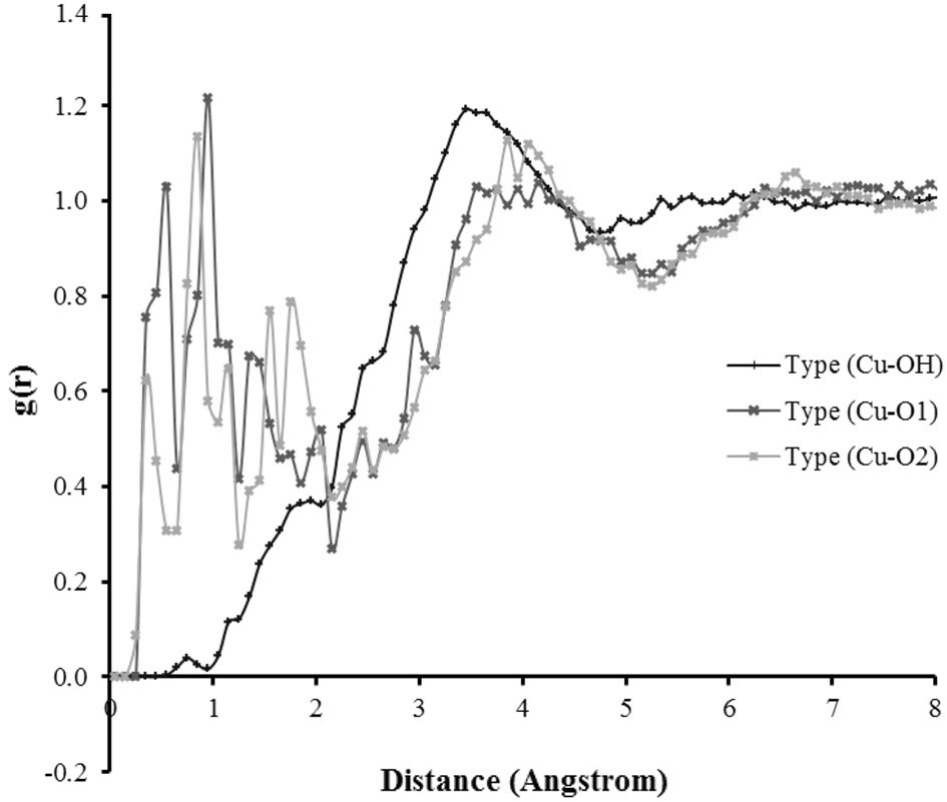
Radial distribution functions of functional group moieties of butyric acid with CuO in a water system.

The functional group attachment chemistry of surface treated nanoclusters on simulation platforms demonstrated three different styles of bonding during the different timespans of simulation; (a) uni-dentate, (b) bi-dentate and (c) bridging attachment. From the simulation perspective, the RDFs shown in Fig. 9 were used to interpret these attachments found on the surface of nanoclusters.

The peaks represented in Fig. 8 show the RDF of Type (Cu-O1) and Type (Cu-O2) at 2.6–2.7 Å that the attachment of the COO^−^ group oxygen to the metal surface is interpreted as a formation of bridging coordination. This is coherent with Raj^36^ statement that Cu–Cu shows a bridging style at 2.6–2.69 Å^37^, which is also known as paddle wheel formation.

**Figure 10:**
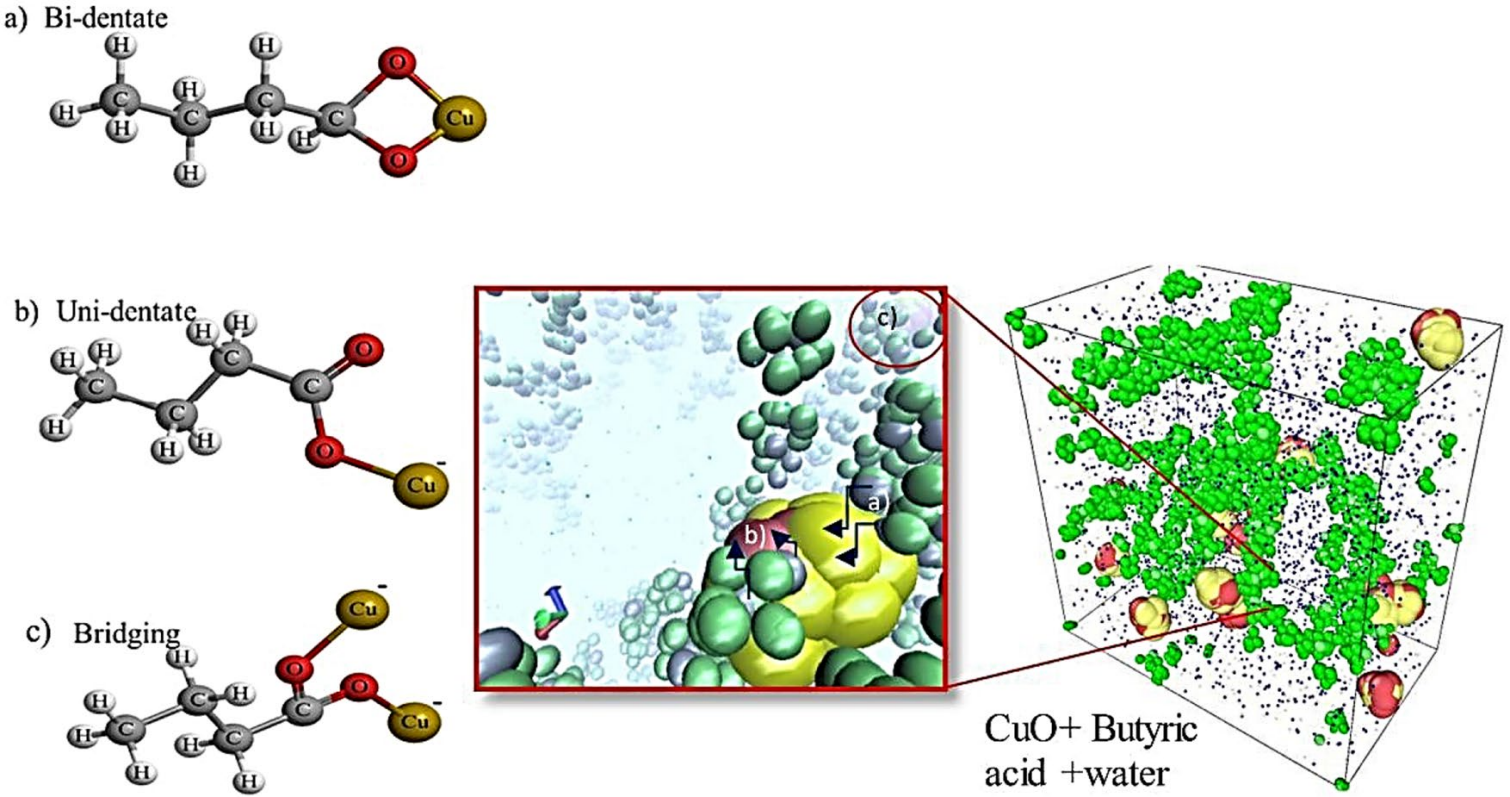
The carboxylate attachment to CuO surface showing different styles of attachment; where point (**a**) represents the predictive bi-dentate, point (**b**) represents the predictive uni-dentate and point (**c**) bridging ligand.

The BA functional group attachment to CuO nanoclusters is reflected by the FTIR spectra showing different chelation coordination formations. The carboxylate attachment to the CuO surface produced a bridging ligand arrangement to two metal centers, which led to formation of a metal–carboxylate bond (Cu–Carboxylate bond) where the bridging arrangement is known as binuclear compounds as shown in Fig. 10.

The phenomenon shown a visualization of a single Cu nanoparticle was surrounded by BA molecular chains in which the polarized parts are connected to the Cu nanoparticles as shown in Fig. 10, at point a, b and c. Moreover, the unidentate and bidentate can be exchangeable accordingly based on the pH and time of the reactions 2 h or 24 h. However, the three attachment styles are shown at different time scales during simulation equilibration.

**Figure 11:**
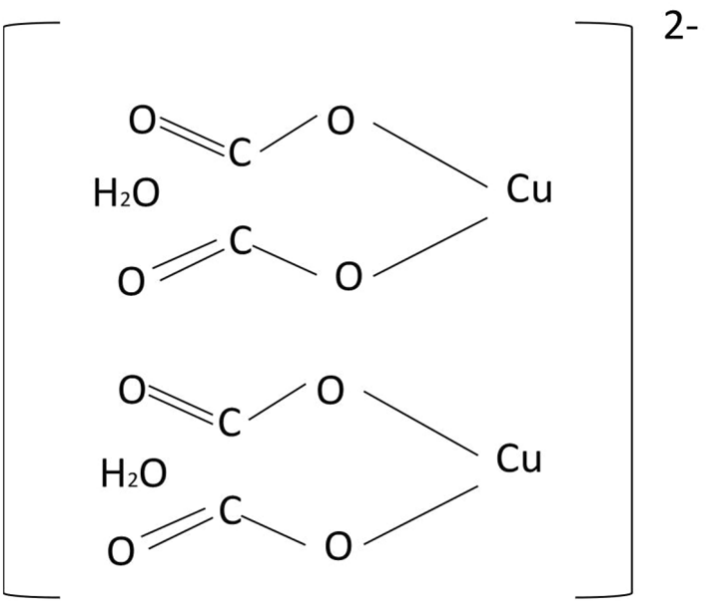
Predicted structure of [2Cu(CO_2_)_2_(H_2_O)]^2−^ formation; reference (CAS Number:55671-32-4).

The nanoparticles demonstrated a combination of Cu bonding to a carboxylate group in water, predictably forming [2Cu (CO_2_)_2_(H_2_O)]^2−^, which is illustrated in Fig. 11. This formation was also predicted and investigated by Raj^36^ with a distance between Cu–O–C as 1.28 Å, and this similar value was also obtained by F. Valach et al.^38^. Furthermore, from the simulation RDF results showed some peaks at 1.28–1.5 Å; these peaks representing moieties attachment to Cu. The two vacancies of C^−^ in Fig. 11 are generally occupied by alkyl group (R) attachments, while the interaction takes place.

The attachment of Cu with a hydroxyl group illustrated by black line in Fig. 8 represents similar metal–ligand correlation as carried out by Nimmermark et al.^39^, where they studied different metal oxides attachment to carboxylate group of acids and hydroxyl moieties of water, the bonding distance was found in between 2.68 Å in the Cu-(OH) group. While in our case the black line shown in Fig. 8, demonstrates the bond distance between 2.7 and 3 Å. This stretch shaft might be due to the use of pair potentials during simulation and random seeding of velocity through the system. Moreover, this stretch shaft is related to harmonic mediations of bonds caused by random forces in the system.

**Figure 12:**
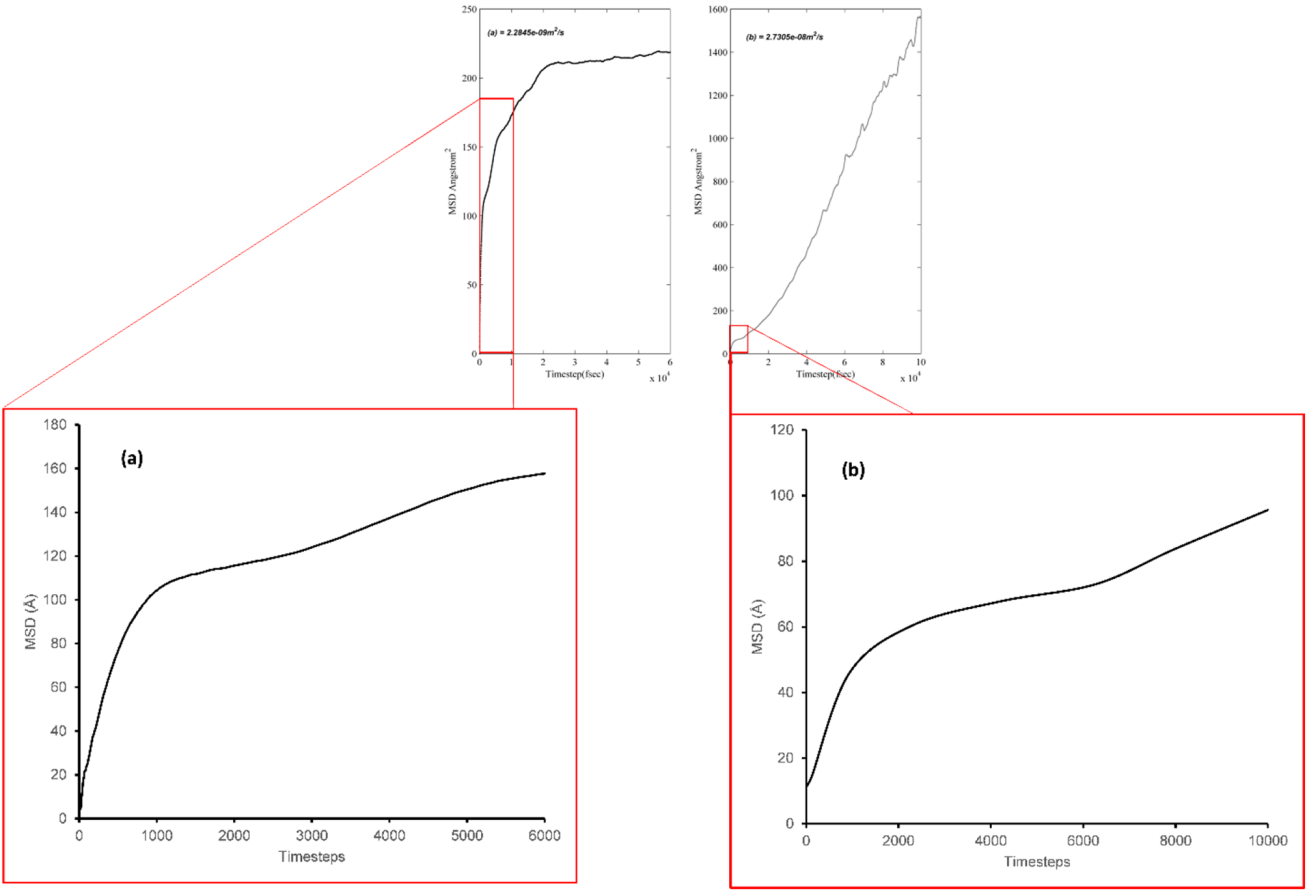
Diffusion coefficients of nanocluster CuO in water-based liquids: (**a**) diffusion coefficients of nanocluster CuO in water with BA (2.28e−09 m^2^/s); (**b**) diffusion coefficients of nanocluster CuO in water (2.73e−08 m^2^/s).

A comparative study of water–CuO nanocluster diffusion to the modified system was conducted and it was found that the modified nanoclusters show low diffusion capabilities as compared to the measured values of the reference system. The 4 nm CuO–water system diffusion coefficient has already been investigated by Loya et al. and it can be concluded that both reference system diffusion coefficients were concurrent; however, they were higher than the modified CuO nanocluster system. Consequently, the analyzed results and their differences helped in depicting a rate of agglomeration and diffusivity between both systems as shown in Fig. 12. Systems with nanoclusters presented a higher diffusion rate than the modified system, because the addition of a functional group changed the viscosity of the system, leading to a change in diffusion parameters.

The obtained results revealed that the three formations of bonding structures were observed during different test and simulation phases. In contrast to experimental work, the simulation was able to further explain the agglomeration spotted by RDF and diffusion coefficient. Future work can be carried out using the correct amount of a functional group with changing of the pH over the simulation platform.

Moreover, for accuracy, multiple slopes were taken for different sets of frames for calculating the diffusion rate and then the average was calculated to analyze the mean diffusion rate as shown in Table 4. Figure 12 shows the expanded version of the timesteps. Figure 12a,b demonstrate expanded timesteps of about one by tenth of the total simulation time. The diffusion rate of BA modified CuO water system is comparatively slower than that of the CuO-water nanofluid. This is because the BA modified CuO-water system shows agglomeration in the diffusion state thereby hampering the diffusivity rate.

**Table 4:** Diffusion rates calculated for different segments of simulation and then average was taken.

Block	303 K BA-CuO-water diffusion blocks (m^2^/s)	303 K Water + CuO diffusion blocks (m^2^/s)
1	2.61E−08	9.86E−09
2	6.75E−09	1.35E−08
3	4.66E−09	2.09E−08
4	2.79E−09	2.68E−08
5	− 5.46E−11	3.47E−08
6	5.83E−10	3.62E−08
7	5.90E−10	2.82E−08
8	− 2.25E−10	3.62E−08
9	4.98E−10	2.20E−08
10	2.36E−10	3.54E−08
Average	4.19E−09	2.64E−08

Second column holds the diffusion rate for BA modified CuO-water system. Third column holds the diffusion rate for CuO-water system.

### Effect of aggregation kinetics on viscosity

**Figure 13:**
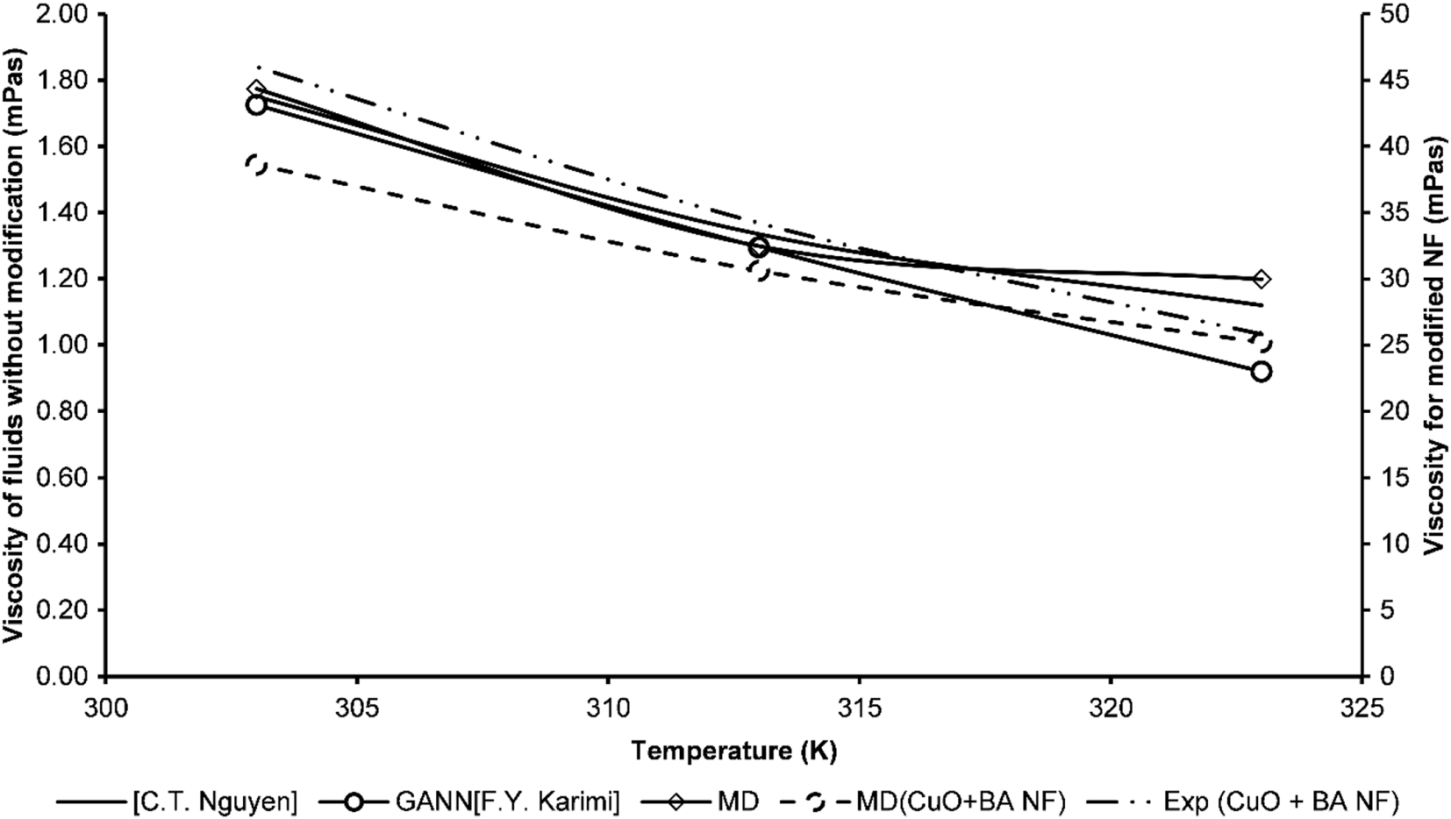
Viscosity of surfactant modified CuO nanofluid and CuO nanofluids.

The aggregation kinetics affect the viscosity of the fluid a lot. As the aggregation happens the fluid starts to show non-Newtonian behavior within the fluid layers. Therefore, it causes drag between the layers. This drag is associated with friction developed between particles and fluid atoms. This friction enhances as particle concentration increases. However, in this study we did not overlook on the change in concertation aspect of the material rather we were more concerned about change in the aggregation kinetics with temperature. Figure 13 demonstrates the change in viscosity of the different nanofluids measured from simulations and experiments. It is found that pure CuO-water nanofluids show viscosities around 0.8–1.2 cp. However, as the surfactant butyric acid is added to the system it shows a drastic increase in viscosity of nanofluid i.e., from 1.2 cp it jumps to 30 cp at average. Moreover, this aggregation has already been discussed earlier using the radial distribution functions. It is observed that the addition of BA induces aggregation in the system. And thereby, causing hindrance in the flow kinetics of the fluid.

Table 5 demonstrates the viscosities attained from various simulations at 313 K. In Table 1 time periods of different simulations are presented. Table 5 also contains values of various viscosities at similar boundary conditions but at different timesteps. Table 5 obtained results verification which were achieved using pressure autocorrelation functions and velocity autocorrelation functions. Stress autocorrelation analysis was also conducted to determine the variation in the stress tensor during simulation.

**Table 5:** Experimental results and the calculated viscosities results are listed at different timeperiods using MDS.

Temperature (K)	Simulations	Dt(fs)	Runs after equilibration	Time(ns)	Sim η (Pa s)	Exp η (Pa s)
313	Sim a	160	25,000	4.00	0.050488	**0.0342**
313	Sim b	160	35,000	5.60	0.042155	
313	Sim c	160	50,000	8.00	0.034541	
313	Sim d	160	60,000	9.60	**0.030694**	
313	Sim e	160	100,000	16.0	0.024587	

Significant values are in bold.

**Figure 14:**
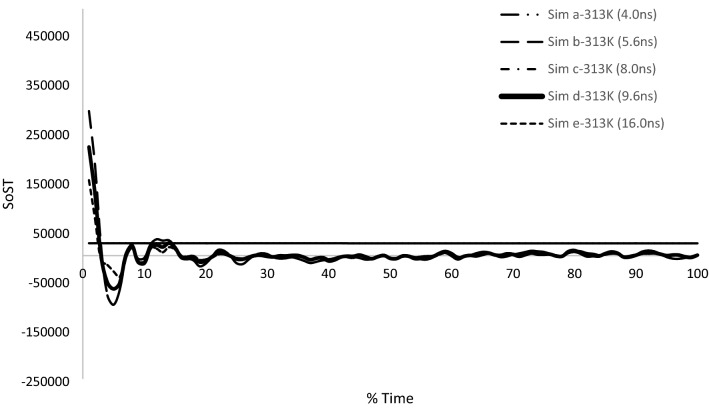
Stress autocorrelation function of different simulation performed at 313 K for BA-CuO H2O nanofluid.

From Fig. 14, the least number of oscillations in stress autocorrelation function is shown by Sim d-313 K as compared to others. However, in the above graph it is hard to correctly identify which simulation stress auto correlation is less oscillatory to depict reliability on value. Therefore, for further examination it is better to utilize the PACF and VACF in conjunction to correctly identify the accurate convergent value.

**Figure 15:**
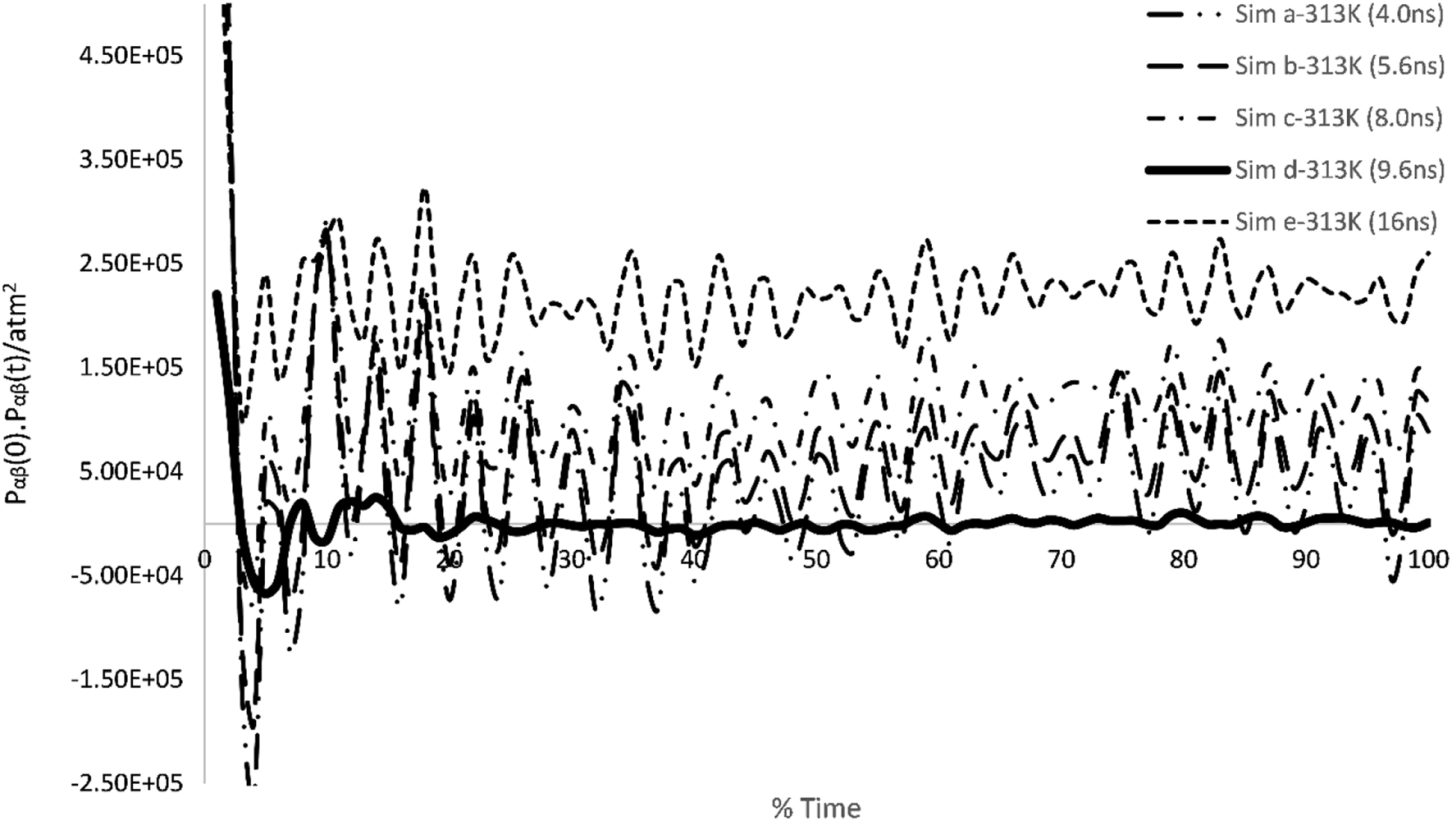
Pressure autocorrelation function of different simulation performed at 313 K for BA-CuO H2O nanofluid.

The Fig. 15 depicts the pressure correlation of various simulations conducted at different timeperiods. Figure 14 is used to quantify the attained reading of viscosity using molecular dynamics simulations. It can be found from the figure that simulation d-313 K shows the least number of oscillations during the performance of simulation. These oscillations were predicted earlier before by Wang et al. for a nanocolloidal solution^40^. They found in their research that, higher oscillations in the pressure autocorrelation function induce high instability in determining the viscosity of nanodispersion. In addition to this, earlier Adil Loya in their doctoral thesis^41^ found the similar behavior regarding pressure correlation and exclaimed that, oscillations cause uncertainty in determining the viscosity of nanofluids. Therefore, keeping in mind the above research, it is well established from Fig. 14 that simulation d-313 K out performed in determining the viscosity of the nanofluid being tested here.

**Figure 16:**
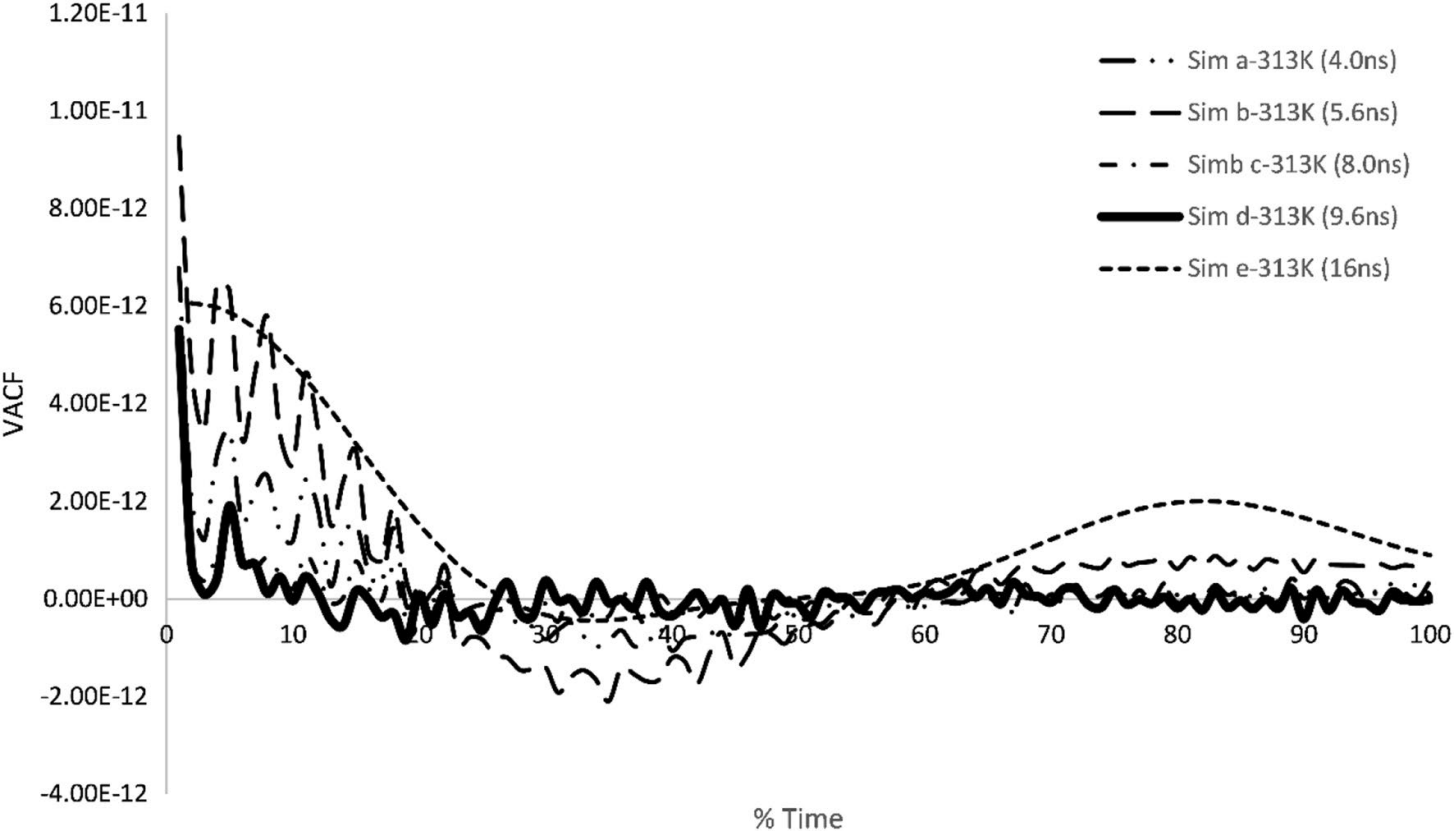
Velocity autocorrelation function of different simulation performed at 313 K for BA-CuO H2O nanofluid.

Moreover, as the PACF defines the reliability over the obtained readings from molecular dynamics simulation in the similar context velocity autocorrelation function (VACF) can give us insight over the dynamical aspect of the system. Figure 16 depicts the VACF of several simulations conducted at various timesteps for 313 K. Over here we can observe that the Simulation d-313 K graph has higher grade of smooth decaying trend as compared to other conducted simulations. However, oscillations within this graph are caused by the low density of the particles as also suggested by Wang et al.^40^. The rest of the other graphs within this figure are demonstrating high levels of oscillations and therefore, showing less reliability over calculated results. For conducting viscosity analysis simulations were conducted for longer time after equilibration time for attaining stability in results.

### Comparison with CuO diffusion in pure water system

As the simulation was carried out for only several picoseconds, the system then started to show high levels of agglomeration due to the surface functionalized BA that tends to create Van der Waal forces of attraction between nanoclusters. Due to this, electrostatic repulsion is decreased, causing the attraction to dominate and a net potential energy barrier is hard to overcome the agglomeration between nanoparticles. This agglomeration is mostly caused by additional surfactants depending on the nature of the surfactant species and the bonding mechanism between nanoparticle and surfactant.

Later, the rate of diffusion extrapolates the working of the nanoclusters diffusion after surfactant attachments. The seizing of the diffusion of metal ions, i.e., Cu^++^ ions in water is benefitted from functional group addition. Moreover, surface treatment of nanoparticles causes the rate of diffusion to decrease as compared to pure nanocluster diffusions. The use of the molecular dynamics for predicting the functional group attachment to the metal oxide can be developed to a scale where these nanoparticles can be simulated in biological fluids. The proposed diffusion rate of CuO and surface treated CuONPs diffused in pure water is in good agreement while comparing them to previous studies in which diffusion rate has been reported 1.15E−8 m^2^/s by Loya et al.^42^.

Moreover, in a later study by Loya et al. they found diffusion coefficients in the range of 1.0E−8 to 2.0E−8 m^2^/s for various concentration of CuO nanoparticles diffused in water^43^. Thereby, the above two examples shows that addition of surfactant to CuO nanoparticles causes agglomeration to which a decrease in diffusion coefficient is recognized from Fig. 11.

### Applications and future prospect of this simulation

The utilization of CuO nanoparticles has been comprehensively discussed in this study, but for future applications, this simulation can further be taken to treat COVID-19/SARS viruses as well. As the applicability of inhibiting viruses using CuONPs has been shown by multiple studies. Moreover, according to Grass et al. CuO ions diffused over bacteria can cause cell damage where this phenomenon is known as “Contact Killing”^44^. However, to underpin the knowledge of how Cu ions interact and make contact for killing viruses, this can be explained profoundly with molecular dynamics. In line with this, conducting such simulations in conjunction with fatty acids for providing further inhibition power can help in excavating more potential of functionalized nanoparticles.

CuONPs can also be utilized for controlled nucleation, a process which occurs in the first stage of crystallization of gas hydrates for determining their nucleation path^45^. It was found by Aliabadi et al. that CuONPs reduce the induction by 97% and provide 34% enhancement in storage capacity of gas hydrates when compared with pure water^45^. However, Aliabadi et al. only conducted experimental study but to get deep insight about gas hydrate nucleation process; Hu et al.^46^ performed MD simulations to ascertain nucleation patterns of methane hydrate molecules, but they were unable to find the cause of methane molecules aggregation. Nevertheless, using MD they discovered that methane hydrate nucleation occurred at some distance from the methane-water junction due to low concentrations of nanobubbles. Loche et al.^47^ explored the evaporation dynamics of chlorine ions in aqueous solution and stated that the hydration of ions should be carried out in a saturated vapor state. Using MD simulations, it was also revealed during long duration evaporation simulations that chloride ions absorb seven water molecules, which is validated using dielectric continuum.

Carbon hydrate samples were collected from the seafloor in China, which were then extracted from deionized water dispersion and then put in investigation for determining CO_2_ kinetics in organic matter by using LNMR technique^48^. It was further explored that the presence of naturally occurring acid and water-dissolvable organic matter, containing mostly lignin and protein/amino sugars could encourage the CO_2_ formation and further aid researchers in separation of CO_2_ hydrates from organic matters. Lu et al.^49^ also investigated the molecular kinetics of gas hydrate in the presence of organic ions (methylene blue) at different temperatures in MD simulation. Based on varying temperature effected the growth rate of CO_2_ hydration and it was suggested that high temperature conditions are more favorable. Moreover, the formation of amorphous clusters can also interrupt the growth rate of hydrates, therefore will prove to be detrimental in the sequestration process. Therefore, various applications of CuONPs show tremendous usage of these particles for nucleation process, antibacterial applications, and energy storage applications. In addition to this, determining insight regarding the interaction using molecular dynamics can further flourish the underseen nature of these nanoparticles.

## Conclusion

Interestingly, the study that is, the attachments of BA represented a surface functionality of CuONPs, which has been predicted from experimental characterization and simulation visualization. The functionalization of the nanoparticles was experimentally analyzed by FTIR results and predominant peaks, i.e., 1667–1609 cm^−1^, 1668–1557 cm^−1^, etc. which showed 3 major types of attachment to the nanoparticles. Those three different attachments were concluded by using peak differences and these were (a) bi-dentate (b) uni-dentate and (c) bridging carboxylate group attachments.

Finally, the experimental details were mimicked using the molecular dynamics simulation using the LAMMPS platform and quite concurrent results were established on a comparison basis with experimental studies. The visual effects of MDS were sound and completely elaborated on the agglomeration effects of nanoclusters as examined experimentally. Furthermore, the RDF results demonstrated successful attachment of functional group moieties to the nanoparticle surface. RDF distance between Cu-(OH) shows bonding distance of approximately 2.68 Å, which is reasonably achieved according to literature estimations. Hence, this study has nurtured the importance of simulating an aggregated system using molecular dynamics simulation for estimating the bonding distance and bonding style. This simulation gives a visual understanding of high agglomeration levels due to surface treatment of nanoparticles using BA in aqueous suspension. Future perspective that can be considered after conducting these molecular dynamic simulations of surface treated CuONPs is that it can be utilized or reacted with latest viruses for investigating inhibition kinetics﻿.

## Data Availability

The raw/processed data required to reproduce these findings cannot be shared at this time as the data also forms part of an ongoing study. Mediafire link will be provided for data.

## Abbreviations

CuO: Copper oxide
CuO NPs: Copper oxide nanoparticles
CuO–H_2_O: Copper oxide–water system
DPD: Discrete particle dynamics
MD: Molecular dynamics
BD: Brownian dynamics
SPH: Smoothed particle hydrodynamics
°C: Degree Celsius
°K: Degree Kelvin
Wm^−^^1^ K^−^^1^: Watt per meter per Kelvin
mPa s: Milli Pascal second
COMPASS: Condensed-phase optimized molecular potential for atomistic simulation studies
d_t_: Timestep size
R_c_: The cutoff
*k*: Boltzmann constant
T: Temperature
LAMMPS: Large-scale atomic/molecular massively parallel simulator
TEM: Transmission electron microscope
λ: Thermal conductivity
η: Viscosity
RDF: Radial distribution function
a.u: Absorbance unit

## References

[1] Pike J, Chan S-W, Zhang F, Wang X, Hanson J (2006). Formation of stable Cu2O from reduction of CuO nanoparticles. Appl. Catal. A.

[2] Reijnders L (2008). Hazard reduction in nanotechnology. J. Ind. Ecol..

[3] Nel A, Xia T, Mädler L, Li N (2006). Toxic potential of materials at the nanolevel. Science.

[4] Carnes CL, Klabunde KJ (2003). The catalytic methanol synthesis over nanoparticle metal oxide catalysts. J. Mol. Catal. A Chem..

[5] Dutta A (2003). Preparation of sol-gel nano-composites containing copper oxide and their gas sensing properties. J. Sol-Gel. Sci. Technol..

[6] Zhu J (2004). Highly dispersed CuO nanoparticles prepared by a novel quick-precipitation method. Mater. Lett..

[7] Karthik R, Harish Nagarajan R, Raja B, Damodharan P (2012). Thermal conductivity of CuO–DI water nanofluids using 3-ω measurement technique in a suspended micro-wire. Exp. Thermal Fluid Sci..

[8] Wu YY, Tsui WC, Liu TC (2007). Experimental analysis of tribological properties of lubricating oils with nanoparticle additives. Wear.

[9] Morales J, Sánchez L, Martín F, Ramos-Barrado JR, Sánchez M (2005). Use of low-temperature nanostructured CuO thin films deposited by spray-pyrolysis in lithium cells. Thin Solid Films.

[10] Vila M, Díaz-Guerra C, Piqueras J (2010). Optical and magnetic properties of CuO nanowires grown by thermal oxidation. J. Phys. D Appl. Phys..

[11] Xiang H (2011). A novel and facile method to prepare porous hollow CuO and Cu nanofibers based on electrospinning. CrystEngComm.

[12] El-Trass A, ElShamy H, El-Mehasseb I, El-Kemary M (2012). CuO nanoparticles: Synthesis, characterization, optical properties and interaction with amino acids. Appl. Surf. Sci..

[13] Li C-C, Chang M-H (2004). Colloidal stability of CuO nanoparticles in alkanes via oleate modifications. Mater. Lett..

[14] Soleimani E, Taheri R (2017). Synthesis and surface modification of CuO nanoparticles: Evaluation of dispersion and lipophilic properties. Nano-Structures & Nano-Objects.

[15] Dianzani C (2006). Cholesteryl butyrate solid lipid nanoparticles inhibit adhesion of human neutrophils to endothelial cells. Br. J. Pharmacol..

[16] Gonçalves P, Araújo J, Pinho M, Martel F (2009). Modulation of butyrate transport in Caco-2 cells. Naunyn-Schmied Arch. Pharmacol..

[17] Wollowski I, Rechkemmer G, Pool-Zobel BL (2001). Protective role of probiotics and prebiotics in colon cancer. Am. J. Clin. Nutr..

[18] Minelli R (2012). Cholesteryl butyrate solid lipid nanoparticles inhibit the adhesion and migration of colon cancer cells. Br. J. Pharmacol..

[19] Leuba KD, Durmus NG, Taylor EN, Webster TJ (2013). Short communication: carboxylate functionalized superparamagnetic iron oxide nanoparticles (SPION) for the reduction of *S. aureus* growth post biofilm formation. Int. J. Nanomed..

[20] Murthy, P. S. *et al. 2011 International Conference on Nanoscience, Engineering and Technology (ICONSET)*, 490–493.

[21] Rada-Iglesias A (2007). Butyrate mediates decrease of histone acetylation centered on transcription start sites and down-regulation of associated genes. Genome Res..

[22] Gutiérrez TJ, Alvarez VA (2018). Handbook of Nanomaterials for Industrial Applications.

[23] Scandura G, Sajjad M, Singh N, Palmisano G, Rodríguez J (2021). On the selectivity of butyric acid photoreforming over Au/TiO2 and Pt/TiO2 by UV and visible radiation: A combined experimental and theoretical study. Appl. Catal. A.

[24] Xue T (2017). Molecular ligand modulation of palladium nanocatalysts for highly efficient and robust heterogeneous oxidation of cyclohexenone to phenol. Sci. Adv..

[25] Graham SP (2021). Antiviral efficacy of metal and metal oxide nanoparticles against the porcine reproductive and respiratory syndrome virus. Nanomaterials.

[26] Borkow G, Zhou SS, Page T, Gabbay J (2010). A novel anti-influenza copper oxide containing respiratory face mask. PLoS ONE.

[27] Xu LJ, Zhao JX, Zhang T, Ren GG, Yang Z (2009). In vitro study on influence of nano particles of CuO on CA1 pyramidal neurons of rat hippocampus potassium currents. Environ. Toxicol. Int. J..

[28] Plimpton S (1995). Fast parallel algorithms for short-range molecular dynamics. J. Comput. Phys..

[29] Sun H, Ren P, Fried JR (1998). The COMPASS force field: Parameterization and validation for phosphazenes. Comput. Theor. Polym. Sci..

[30] Sun H (1998). COMPASS: An ab initio force-field optimized for condensed-phase applications overview with details on alkane and benzene compounds. J. Phys. Chem. B.

[31] Zhao L, Liu L, Sun H (2007). Semi-ionic model for metal oxides and their interfaces with organic molecules. J. Phys. Chem. C Nanomater. Interfaces.

[32] Gingold RA, Monaghan JJ (1977). Smoothed particle hydrodynamics: Theory and application to non-spherical stars. Mon. Not. R. Astron. Soc..

[33] Jiang W (2010). Effect of sodium oleate as a buffer on the synthesis of superparamagnetic magnetite colloids. J. Colloid Interface Sci..

[34] Zhang L, He R, Gu H-C (2006). Oleic acid coating on the monodisperse magnetite nanoparticles. Appl. Surf. Sci..

[35] Huang X (2007). Self-assembled virus-like particles with magnetic cores. Nano Lett..

[36] Raj G (2010). Advanced Inorganic Chemistry: Vollume II.

[37] Nockemann P, Thijs B, Parac-Vogt TN, Van Hecke K, Van Meervelt L, Tinant B, Hartenbach I, Schleid T, Ngan VT, Nguyen MT, Binnemans K (2008). Carboxyl-functionalized task-specific ionic liquids for solubilizing metal oxides. Inorg. Chem..

[38] Valach F, Melník M, Bernardinelli G, Fromm K (2006). A structural study of copper(II) carboxylates: Crystal structure and physical characterisation of [Cu2(2-bromopropanoato)4(caffeine)2]. J. Chem. Crystallogr..

[39] Nimmermark A, Öhrström L, Reedijk J (2013). Metal-ligand bond lengths and strengths: Are they correlated? A detailed CSD analysis. Z. für Kristallogr. Cryst. Mater..

[40] Wang T, Wang X, Luo Z, Cen K (2008). Physics behind the oscillation of pressure tensor autocorrelation function for nanocolloidal dispersions. J. Nanosci. Nanotechnol..

[41] Loya, A. *Large Scale Dynamic Molecular Modelling of Metal Oxide Nanoparticles in Engineering and Biological Fluids* (2015).

[42] Loya A, Stair JL, Ren G (2014). The study of simulating metaloxide nanoparticles in aqueous fluid. Int. J. Eng. Res. Technol..

[43] Loya A, Ren G (2015). Molecular dynamics simulation study of rheological properties of CuO–water nanofluid. J. Mater. Sci..

[44] Grass G, Rensing C, Solioz M (2011). Metallic Copper as an Antimicrobial Surface. Appl. Environ. Microbiol..

[45] Aliabadi M, Rasoolzadeh A, Esmaeilzadeh F, Alamdari A (2015). Experimental study of using CuO nanoparticles as a methane hydrate promoter. J. Nat. Gas Sci. Eng..

[46] Hu W (2022). Three-body aggregation of guest molecules as a key step in methane hydrate nucleation and growth. Commun. Chem..

[47] Loche P, Bonthuis DJ, Netz RR (2022). Molecular dynamics simulations of the evaporation of hydrated ions from aqueous solution. Commun. Chem..

[48] Liu Y (2021). Behaviors of CO2 hydrate formation in the presence of acid-dissolvable organic matters. Environ. Sci. Technol..

[49] Lu Y (2022). Molecular behavior of CO2 hydrate growth in the presence of dissolvable ionic organics. Chem. Eng. J..

